# Laboratory Surge Response to Pandemic (H1N1) 2009 Outbreak, New York City Metropolitan Area, USA

**DOI:** 10.3201/eid1601.091167

**Published:** 2010-01

**Authors:** James M. Crawford, Robert Stallone, Fan Zhang, Mary Gerolimatos, Diamanto D. Korologos, Carolyn Sweetapple, Marcella de Geronimo, Yosef Dlugacz, Donna M. Armellino, Christine C. Ginocchio

**Affiliations:** North Shore–Long Island Jewish Health System Laboratories, Manhasset, New York, USA (J.M. Crawford, R. Stallone, F. Zhang, M. Gerolimatos, D.D. Korologos, C.C. Ginocchio); Krasnoff Quality Management Institute of the North Shore–Long Island Jewish Health System, Manhasset (C. Sweetapple, M. de Geronimo, Y. Dlugacz); North Shore University Hospital, Manhasset (D.M. Armellino)

**Keywords:** Influenza A pandemic (H1N1) 2009, influenza, surge capacity, pandemic influenza, bioterrorism and preparedness, viruses, synopsis, expedited, New York, USA

## Abstract

The North Shore–Long Island Jewish Health System Laboratories serve 15 hospitals and affiliated regional physician practices in the New York City metropolitan area, with virus testing performed at a central reference laboratory. The influenza A pandemic (H1N1) 2009 outbreak began in this area on April 24, 2009, and within weeks respiratory virus testing increased 7.5 times. In response, laboratory and client service workforces were increased, physical plant build-out was completed, testing paradigms were converted from routine screening tests and viral culture to a high-capacity molecular assay for respiratory viruses, laboratory information system interfaces were built, and same-day epidemiologic reports were produced. Daily review by leadership of data from emergency rooms, hospital facilities, and the Health System Laboratories enabled real-time management of unfolding events. The ability of System laboratories to rapidly increase to high-volume comprehensive diagnostics, including influenza A subtyping, provided key epidemiologic information for local and state public health departments.

## Abstract 2

Emergency preparedness programs are critical.

Local sentinel laboratories are a critical component of the Laboratory Response Network, providing frontline diagnostics and, in many instances, the initial reporting for infectious disease outbreaks. In the event of a major health crisis, the responses of a regional clinical laboratory can be central to the ability of civic authorities and healthcare systems to handle such emergencies. This report describes successful steps taken by a hospital-based regional reference laboratory in response to a 7.5× increase in respiratory virus testing during the first 3 weeks (April 27–May 15, 2009) of an outbreak of a novel influenza A (H1N1), now referred to as influenza A pandemic (H1N1) 2009, in the greater New York City metropolitan area.

The North Shore–Long Island Jewish Health System (NSLIJHS) is the third largest nonsectarian not-for-profit health system in the United States and serves Nassau and Suffolk Counties, New York, and the Queens and Staten Island boroughs of New York City. The NSLIJHS Central Laboratories serve 15 hospitals and affiliated regional physician practices; virus testing is performed at the Centralized Laboratories in the Clinical Virology Laboratory (CVL). In the aftermath of the anthrax event of September 2001, NSLIJHS developed an extensive system-wide emergency preparedness plan to deal with potential biothreat and bioterrorism events that could greatly affect a health system. This plan was tested, beginning Friday evening, April 24, 2009, when 20 students 14–17 years of age with symptoms of an influenza-like illness sought evaluation at the pediatric emergency room of Schneider Children’s Hospital at Long Island Jewish Medical Center, one of the hospitals served by NSLIJHS. The students were among those attending a preparatory school in Queens, New York, who began experiencing influenza-like symptoms April 22–23, 2009 (*1*). Some students had recently traveled to Mexico, raising immediate concern about pandemic (H1N1) 2009 (*2*). The following day, Saturday, April 25, an additional 67 persons 11–18 years of age and 16 children <8 years of age were evaluated in NSLIJHS emergency rooms. Over the next 3 days, influenza-related cases at NSLIJHS emergency rooms increased rapidly. Specimens from index patients at Long Island Jewish Medical Center on April 24–25 were screened for influenza A/B antigen, using the 3M Rapid Detection Flu A+B test (3M Medical Diagnostics, St. Paul, MN, USA). In conjunction with the New York City Department of Health (DOH), the Medical Center shipped 35 specimens with test results positive for influenza A to the Centers for Disease Control and Prevention (CDC). Testing at CDC confirmed that 28 of the 35 influenza A–positive samples were influenza A pandemic (H1N1) 2009 (*1*). The remaining 68 samples from the Long Island Jewish Medical Center (i.e., those with negative rapid test results) were referred to CVL for direct fluorescent antibody (DFA) testing and viral culture, according to the usual protocol.

On Monday, April 27, 2009, an emergency operations status was declared for NSLIJHS. Herein, we detail the specific steps taken to increase the surge capacity at the NSLIJHS Central Laboratories, thereby enabling timely reporting of respiratory virus test results.

## Standard Testing for Respiratory Viruses and Test Capacity

During the normal influenza season, the clinical laboratories of NSLIJHS hospitals perform rapid influenza A+B antigen testing, using either BinaxNOW A+B test (Inverness, Scarborough, MA, USA) or the 3M test. Nasopharyngeal swab samples in Universal Transport Media (Diagnostic Hybrids Inc., Athens, OH, USA) and nasopharyngeal wash and aspirate samples are tested. Specimens with test results positive for influenza A or B are not processed further unless warranted by underlying patient conditions. Due to the suboptimal sensitivity of rapid antigen tests, all samples with negative test results are forwarded to CVL for detection of adenovirus, human metapneumovirus, influenza A and B, parainfluenza viruses 1, 2, and 3, and respiratory syncytial virus by DFA testing, using D3 Ultra Respiratory Virus reagents (Diagnostic Hybrids Inc.) and by rapid respiratory virus culture, using R-Mix cells (Diagnostic Hybrids Inc.).

NSLIJHS laboratories routinely encounter a seasonal increase in respiratory virus testing, peaking in mid-to-late February and waning by May. The historic maximum test volume occurred in February 2008, when CVL tested 6,021 samples and clinical laboratories system-wide performed 2,901 rapid influenza tests, for a combined daily average of 308 tests. During April 1–23, 2009, CVL tested 1,955 samples and clinical laboratories system-wide performed 676 rapid influenza tests, for a combined daily average of 119 tests. These volumes were similar to those for preceding years.

## Molecular Detection of Respiratory Viruses

During 2008, the central NSLIJHS Molecular Diagnostics Laboratory performed extensive validation studies of the Luminex xTAG Respiratory Virus Panel (RVP) assay (Luminex Molecular Diagnostics, Toronto, Canada) (*3*,*4*). The version of the RVP assay that has been cleared by the US Food and Drug Administration detects adenovirus, human metapneumovirus, parainfluenza viruses 1, 2, and 3, rhinovirus/enterovirus group, respiratory syncytial virus, and influenza A and B. This RVP assay can subtype influenza A as seasonal human H1 or H3 virus. The research-use-only version of the RVP assay also detects parainfluenza 4 and coronaviruses OC43, NL64, 229E, and HKU-1. Prior to the outbreak of pandemic (H1N1) 2009, the RVP assay was used for selected clinical cases and research studies, with the intention of converting to use of the RVP assay for all respiratory virus testing during the off-peak 2009 summer months.

## Laboratory Testing during the Novel Influenza (H1N1) Outbreak

**Figure 1:**
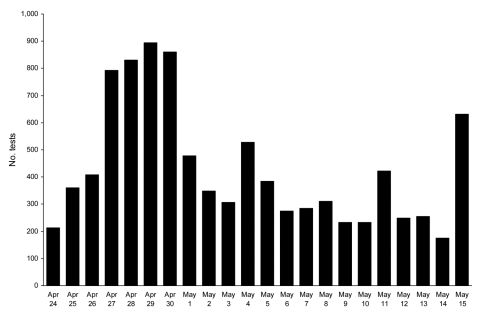
Daily clinical virology test volumes in the North Shore–Long Island Jewish Health System, New York City metropolitan area, USA, April 24–May 15, 2009. General clinical laboratories performed influenza A/B rapid antigen testing only. The central Clinical Virology Laboratory performed direct immunofluorescence antibody testing and R-Mix viral culture, and beginning May 2, the central Molecular Diagnostics Laboratory performed molecular testing for respiratory viruses (xTAG Respiratory Virus Panel, Luminex Molecular Diagnostics, Toronto, Ontario, Canada).

By Monday, April 27, 2009, it was clear that an unusual event was occurring (*5*,*6*). Rapid influenza testing at all NSLIJHS clinical laboratories and the centralized laboratories increased dramatically (Figure 1). On April 29, daily test volumes peaked at 903 tests, representing a 7.5× increase over the prior average daily volume for April and a sustained 3× increase over the February 2008 record daily volume of 308 tests.

The weekend of April 25–26, prior to declaration of a system-wide emergency, scheduled CVL staff responded with a round-the-clock effort to keep up with testing needs. On April 27, despite full staffing, considerable overtime was required to perform testing. On April 28, a command meeting was held at the NSLIJHS central laboratories to delineate an action plan to respond to the crisis. The major issues were staffing, testing algorithms, laboratory space, laboratory information systems and biostatistical reporting, leadership roles, and client service functions. The action plan was immediately authorized by system leadership. A key consequence of the plan was immediate deployment of an enhanced and diversified work force, including nonlicensed support staff, licensed research staff, laboratory information services personnel, and biostatistical reporting staff. Beginning April 28, these actions enabled CVL to expand its weekday working hours from 6:00 am–6:00 pm to 5:00 am–1:00 am and its weekend working hours from 8:00 am–4:00 pm to 6:00 am–10:00 pm. With this personnel strategy, CVL was able to report DFA assay and R-Mix culture results on a real-time basis throughout the crisis. To accommodate the high volume of R-Mix cultures, CVL reduced the normal test algorithm of screening at 24 hours, 48 hours, and 7 days to a single screening and confirmatory testing at 48 hours. Under these unusual circumstances, this change was considered acceptable as historic laboratory data had demonstrated that 97%–98% of all respiratory viruses are detected in 48 hours.

A second key element of the April 27–May 1 work week was the initiation of RVP testing for influenza A subtyping. The assay was needed to 1) identify which patients were possibly infected with virus subtype H1N1 rather than circulating seasonal H1 or H3 strains and 2) track the magnitude of the outbreak. By May 1, in consultation with the New York State and New York City DOHs, the NSLIJHS Molecular Diagnostics Laboratory began testing, with the RVP assay, the remaining samples for index patients screened on April 24–25 (i.e., the 68 archived samples with negative rapid influenza A/B antigen screening results) as well as all incoming samples with test results positive for influenza A by DFA assay and/or culture.

Due to the large volume of incoming and archived samples, RVP testing was prioritized for hospitalized patients, followed by persons known to be at risk as a result of the school exposure or recent travel to Mexico. May 2–3, the laboratory identified, by RVP assay, 141 samples with nonsubtypeable influenza A, 78 samples with seasonal virus subtype H3, and 2 samples with seasonal virus subtype H1. Including the initial 28 samples that were sent to CDC, the NSLIJHS Molecular Diagnostics Laboratory had identified 169 confirmed or probable pandemic (H1N1) 2009 cases. On May 5, 101 samples with nonsubtypeable influenza A were tested by the New York State Wadsworth Center Laboratory of Viral Diseases: 99 had test results positive for pandemic (H1N1) 2009 and 2 had inconclusive test results due to low virus titers (*7*). These data indicated that during the outbreak, the predictability of a nonsubtypeable influenza A virus identified by RVP assay to be pandemic (H1N1) 2009 was high (*7*). In July 2009, the NSLIJHS Molecular Diagnostics Laboratory obtained New York State approval to confirm cases of pandemic (H1N1) 2009 by using the published CDC method. The ability to subtype influenza A, ruling out seasonal subtypes H1 and H3, and to detect additional respiratory viruses by RVP assay (*8*) within 24 hours enabled NSLIJHS leadership to know whether high-risk patients, inpatients, or members of the System’s workforce had probable pandemic (H1N1) 2009.

From that point forward, the Molecular Diagnostics Laboratory provided RVP subtyping results (seasonal H1, H3, or nonsubtypeable) within 24 hours for critically important cases identified by infection-control or civic authorities, especially health officials making public health decisions about regional school systems, and within 48–72 hours for lower priority cases. The laboratory documented that the sensitivity of the RVP assay for detecting all influenza A types was far superior to that for other test methods (*8*), justifying RVP testing for admitted patients with negative influenza A/B rapid test results. This simplified protocol was instituted May 11, 2009, in consultation with system and regional civic authorities.

**Figure 2:**
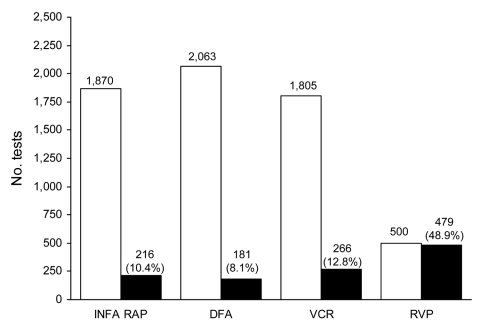
Cumulative virology test volumes and influenza A–positive results, North Shore–Long Island Jewish Health System, New York City metropolitan area, USA, April 24–May 15, 2009. INFA RAP, rapid antigen test for influenza A; DFA, direct immunofluorescent antibody test; VCR, rapid respiratory virus culture by R-Mix (Diagnostic Hybrids Inc., Athens, OH, USA); RVP, Luminex xTAG Respiratory Virus Panel (Luminex Molecular Diagnostics, Toronto, Canada). White bars, number of tests with negative results for influenza A.; black bars, number of test results positive for influenza A. Actual numbers are included above the bars. For influenza A, the percentages of samples positive for influenza A are shown in parentheses.

A detailed scientific analysis of the virology of the outbreak, especially the sensitivities and specificities of the tests, is described elsewhere (*8*); for this publication, summary results are given. Figure 2 shows the total number of positive and negative RVP influenza A test results during April 24–May 15, 2009. Of the total 979 RVP test results, 320 were negative and 677 were positive for any identifiable respiratory virus. A variety of viruses were identified in the 677 samples, including nonsubtypeable influenza A (345 samples), seasonal influenza A subtype H3 (126 samples), seasonal influenza A subtype H1 (5 samples), influenza B (3 samples), rhinovirus/enterovirus group (112 samples), human metapneumovirus (24 samples), parainfluenza viruses 1–4 (40 samples), adenovirus (9 samples), coronaviruses (8 samples), and respiratory syncytial virus (5 samples). Multiple viruses were identified in 41 patients. The outbreak began to subside at the end of June 2009; 8,766 rapid influenza A tests, 8,754 rapid influenza B tests, 8,858 DFA assays, 5,786 viral cultures, and 4,853 RVP assays (36.9% with nonsubtypeable influenza A results) had been performed. This finding represented a total of 34,017 tests for 11,624 patients, a volume that would normally equal the amount of testing performed over a 1-year period.

## Physical Plant Construction

The decision was made on April 28 to immediately expand the CVL into contiguous space. Although the severity and duration of the outbreak were unknown, failure to be proactive in expanding the surge capacity of the laboratory was unacceptable. Over 4 days, a negative-pressure laboratory was completed; biohazard hoods, vacuum, and CO_2_ lines were installed; incubators and ancillary equipment were ordered; and a specimen-processing area with computer terminals was completed. The additional laboratory space enabled a sustained higher RVP testing capacity and was well-justified because the laboratory processed 700–970 tests a day during the second wave of the outbreak (May 15–31).

## Laboratory Information System and Biostatistical Reporting

The Laboratory Information System team accomplished the following tasks over 3 days (April 28–30): 1) created 2 new tests (RVP, novel H1N1 confirmatory) and 3 Laboratory Information System environments (Cerner Classic and Cerner Millennium [Cerner Corp., Kansas City, MO, USA] and Meditech [Medical Information Technology, Inc., Westwood, MA, USA]); 2) validated tests and billing for 11 health information systems; 3) set up a CVL workstation dedicated to influenza specimens; 4) reported daily to infection-control and senior system leadership; 5) reported to the New York State DOH Electronic Clinical Laboratory reporting system; and 6) established logic rules to automatically print positive test results to Client Services. The NSLIJHS Krasnoff Quality Management Institute provided daily biostatistical reports to system leadership. Data assembly was automated by building an Oracle database with interfaces to the Laboratory Information System, with crosschecking to confirm the accuracy and validity of data.

## Leadership and Ancillary Personnel

The medical director of CVL and the Molecular Diagnostics Laboratory (C.C.G.), the overall director of System laboratories (J.M.C.), and the chief operating officer of System laboratories (R.S.) divided responsibilities for oversight of staffing, physical plant resources, supplies, the laboratory information system, courier services, and finance; for daily briefings of laboratory staff, medical staff, infection control, and System leadership; and for DOH notification. This included daily system-wide conference calls at 7:00 am, 3:00 pm, and 11:00 pm. A critical focus was the protection of the healthcare workforce and inpatients from nosocomial spread of pandemic (H1N1) 2009. The medical director reported regularly to the New York City, New York State, and Nassau and Suffolk County DOHs, keeping civic officials apprised of the epidemiology of the outbreak.

An additional key element of the laboratory response was communication with physicians and patients. From April 27 through May 15, the central laboratories’ Client Services, in addition to their usual 8,000–9,000 calls a week, handled an additional 1,000 telephone calls a week pertaining specifically to the outbreak. Laboratory leadership provided scripts to Client Services, including answers to frequently-asked questions. Client Services made strategic calls to physician offices to provide updates on testing protocols and priorities. In addition, the NSLIJHS sales force was redirected to support physician offices, including communication of protocols and procedures and deliveries of supplies.

## Discussion

The fundamental role of a clinical laboratory is to provide medical care to the patient population it serves. The ability to respond to a specific crisis also provides critical support to civic agencies. Although our laboratory did not perform surge testing for the DOHs, nor was testing specifically delineated between our laboratory and the DOHs, our ability to provide comprehensive virus testing, including influenza A subtyping, for such a large patient base (≈6.5 million persons) indirectly assisted the DOHs by providing key diagnostic information with which the DOHs could make management and testing decisions.

The steps taken by our laboratory were strongly supported by system leadership and enabled NSLIJHS to successfully meet this crisis. It is our hope that delineation of these steps will be valuable to other health systems and their laboratories because there undoubtedly will be future public health crises that will demand an immediate increase in reference laboratory testing capacity. The toll of this crisis was on NSLIJHS laboratory personnel. The long hours required to meet testing demands were keenly felt in the first days, when the emergency response had not been activated. However, initial implementation of the emergency response only enabled us to keep up with the crisis, not go beyond it. Routine personnel worked extended hours, despite the support of cross-covering personnel. One key reason was the high level of expertise required to perform the virology and molecular testing; substitute personnel could not be deployed on short notice. The other key reason was the staff’s dedication and their reluctance to go off-duty. Management created obligatory off-duty rotations to ensure our work force was as rested as possible.

The major surge response stratagems, established at the outset, guided NSLIJHS management actions throughout the crisis. Workforce management was top priority. Coordinating the System’s general laboratories with CVL, daily reporting of test volumes and results, and providing support to Client Services enabled the laboratories to remain in synchrony with emergency departments, hospital facilities, and physician practices. Also imperative was the need for all NSLIJHS laboratories to maintain normal operations. At no time during a crisis can normal laboratory services undergo degradation.

Preparedness for infectious outbreaks has increasingly been a point of concern, owing to the threats of bioterrorism and natural diseases. Attention is given primarily to the hypothetical preparedness of first responders and acute-care facilities, either in the form of surveys (*9*–*12*), workflow analysis (*13*), or reviews (*12*,*14*,*15*). The outbreak of severe acute respiratory syndrome in 2003 generated reports from an actual global outbreak. In Hong Kong and Toronto, note was made of the frustrations arising from limited access to laboratory testing, resulting in a decreased ability to provide timely screening of patients for severe acute respiratory syndrome (*16*). The ability of a laboratory to deploy molecular respiratory virus testing was felt to be key for a successful response to an infectious disease outbreak, as such testing is highly sensitive, specific, and capable of high-throughput (*12*).

We believe that there will be future infectious outbreaks that will strain the standing capacity of clinical laboratories, requiring effective implementation of surge capacity responses independent of public health laboratory support. We believe that the steps taken by NSLIJHS laboratories during the initial outbreak of pandemic (H1N1) 2009 and the lessons learned (Table) from that experience are of value. The exceptional 2-way interaction between the NSLIJHS Laboratories and the NY public health laboratories was an excellent example of how sentinel laboratories function as a key component of the Laboratory Response Network system and can serve as a major support for public health in the time of crisis.

**Table:** Lessons learned during clinical laboratory response to pandemic (H1N1) 2009, New York City metropolitan area, USA, April 24–May 15, 2009*

The following were critical to an effective laboratory response:
1. Early assessment and decisive and immediate response by management to laboratory needs
Includes needs related to staffing, supplies, the LIS, physical plant, client relations, and local and state reporting requirements
2. Management of staffing needs
Plans for immediate cross-coverage by trained technical and nontechnical staff
3. Coordination of system general laboratories
Standardization of testing algorithms and prioritization of courier delivery to central clinical virology and Molecular Diagnostics Laboratories
4. Enhanced reporting
Verification of LIS operations for patient-based reporting
Communication to treating physicians
Daily epidemiology reports for System leadership, Infection Control, and hospital administrations
Daily contact with local civic health officials
5. Enhanced client services
Increase number of staff to communicate results and respond to incoming calls, including scripted responses to frequently-asked questions
Maintenance by sales staff of specimen-collection supplies and communication of guidelines for specimen procurement and testing to outreach physician practices
6. Public relations oversight
Communications to news agencies were restricted to the System’s public relations office

*LIS, Laboratory Information Systems; System, North Shore–Long Island Jewish Health System.
